# Gene expression rearrangements denoting changes in the biological state

**DOI:** 10.1038/s41598-021-87764-0

**Published:** 2021-04-19

**Authors:** Augusto Gonzalez, Joan Nieves, Dario A. Leon, Maria Luisa Bringas Vega, Pedro Valdes Sosa

**Affiliations:** 1 University of Electronic Science and Technology, 610051 Chengdu, People’s Republic of China; 2 Institute of Cybernetics, Mathematics and Physics, 10400 Havana, Cuba; 3 Faculty of Physics, University of Havana, 10400 Havana, Cuba; 4 University of Modena and Reggio Emilia, 41125 Modena, Italy; 5 Cuban Neurosciences Center, 11600 Havana, Cuba

**Keywords:** Gene expression analysis, Data processing, Power law, Computational models

## Abstract

In many situations, the gene expression signature is a unique marker of the biological state. We study the modification of the gene expression distribution function when the biological state of a system experiences a change. This change may be the result of a selective pressure, as in the Long Term Evolution Experiment with *E. Coli* populations, or the progression to Alzheimer disease in aged brains, or the progression from a normal tissue to the cancer state. The first two cases seem to belong to a class of transitions, where the initial and final states are relatively close to each other, and the distribution function for the differential expressions is short ranged, with a tail of only a few dozens of strongly varying genes. In the latter case, cancer, the initial and final states are far apart and separated by a low-fitness barrier. The distribution function shows a very heavy tail, with thousands of silenced and over-expressed genes. We characterize the biological states by means of their principal component representations, and the expression distribution functions by their maximal and minimal differential expression values and the exponents of the Pareto laws describing the tails.

## Gene expression markers and distribution functions

Present day technologies allow to measure gene expression (GE) levels in individual cells^1^. By means of techniques of dimensional reduction, such as principal component analysis (PCA)^2–4^, one can show that the GE signature is a good marker of the cell state. Different options for the cell fate, for example, are seen to be resolved as disjoint regions in GE space^5–7^.

With regard to tissues, although conceptually more complex because GE measurements in a small sample contain many different contributions, the procedure has proven its value, for example, in establishing spatial maps of the brain^8^, in order to discriminate between a normal tissue and a tumor^9^, etc. We believe that the GE signature could also be a good marker for the microstate of a tissue portion or sample, which takes account of the different cells entering the sample and the complex signaling system regulating the microenvironment.

In our paper, GE data is analyzed. The data comes from a long-term evolution experiment (LTEE) with *E. Coli* cultures^10^, the Allen Institute study of aging and dementia^11,12^, and The Cancer Genome Atlas (TCGA)^13^. In all of these experiments there are two well defined conditions: an initial or normal state, and a final or disease state. The evolution from initial to final states is precisely defined in the controlled experiment with bacteria. In the other two cases, however, the progression is not documented. Data from normal and disease samples is available and for their analysis we should assume a kind of ergodic hypothesis^14^, stating that the microstates surveyed by the time evolution of a single sample, as time becomes large enough, coincide with those measured from many different samples at a given time.

We use PCA in order to characterize the systems states in GE space. In addition, we study how the GE distribution function is rearranged as the biological state transit from the initial (normal) to the final (disease) state.

Gene transcription, as any process in a living organism, is a noisy process^15^ in which many small elements participate. The general result is a GE distribution function with a heavy tail^16^, of power-like (Pareto) form^17^.

We study how the expressions of all genes are redistributed as the biological state changes from initial to final or normal to disease. By geometric averaging over initial samples in order to smooth down the noise, we compute reference values for each gene in the initial state, $e_{ref}$. Differential expressions are defined as $d = e/e_{ref}$ and the distribution function in the final state is computed. More precisely, we compute integral or cumulative distribution functions. That is, in the over-expression branch we count the number of genes with differential expressions greater than or equal to a given *d*. In the under-expression branch, we count the number of genes with differential expressions lower than or equal to a given *d*. A $d \approx 1$ means that the expression level of a gene has not changed, whereas $d \gg 1$ or $d \ll 1$ correspond to over-expressed or under-expressed (silenced) genes, respectively.

In the studied samples, we found two kinds of GE rearrangements after a change in the biological state. In the first case, most genes take values near the reference ones, and only a small fraction of genes take significant differential expression values. The distribution function is rapidly decaying as *d* departs from 1. Because of the Pareto character, the decay law is $1 / d^{\upsilon }$, with a relatively large value for the exponent. This situation corresponds to relatively close initial and final states, and a “continuous” transition.

The second general case, on the other hand, is characterized by radical expression rearrangements and heavy tails in the distribution functions (small exponents), involving thousands of differentially expressed genes. It corresponds to initial and final states far apart in GE space, and a “discontinuous” transition.

In the next section, we use an analogy with physics in order to build up an intuition with regard to these two kinds of transitions.

## Continuous and discontinuous transitions in Physics

**Figure 1:**
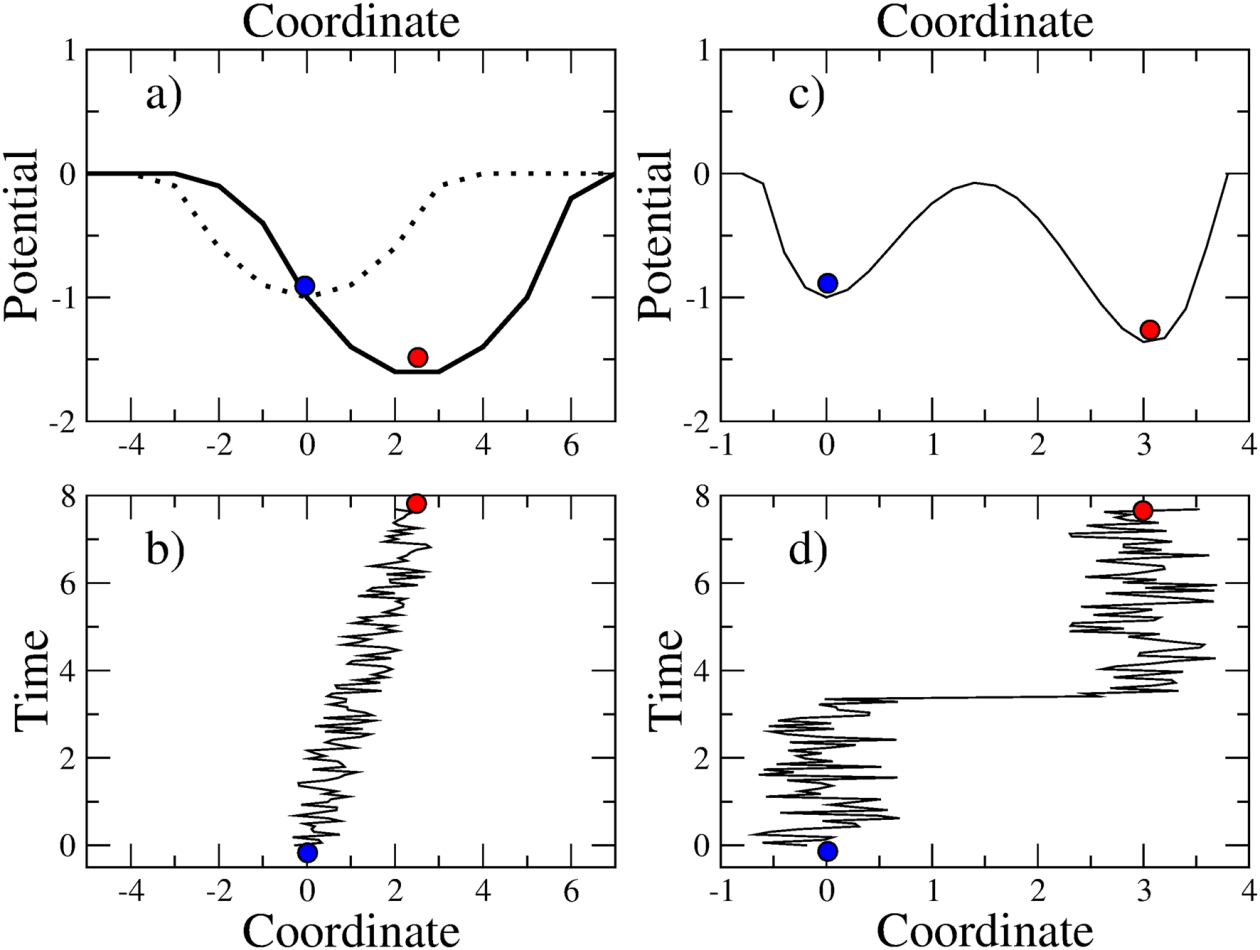
**(a)** and **(b)** Illustration of a continuous transition. The addition of a small electric field to a harmonic potential well causes a modification of the minimum from $\langle x_1 \rangle = 0$ (blue circle) to a nonzero value (red circle). **(c)** and **(d)** Illustration of a discontinuous transition in a double well with distant minima. Random fluctuations may drive a particle, initially in the left well, towards the right deepest well. The barrier separating the two minima should be surpassed.

In Fig. 1a, we draw a nearly harmonic potential well (dashed line). Under the action of a small amplitude noise, the motion of a particle in the well is characterized by a mean value for its position $\langle x \rangle = 0$, corresponding to the potential minimum. This abstract picture may represent a biological system. The x-axis is a coordinate in GE space, and the y-axis is the fitness with a minus sign, such that the minimum of the potential is the state with maximal fitness.

Now, a small amplitude electric field is applied in the *x* direction. The resulting effective potential is drawn with a continuous line. A non zero minimum emerges. As time evolves, the result of the noisy motion is a mean position displacement from $\langle x \rangle = 0$ to the new potential minimum. In the biological analogy, the electric field may be interpreted as a change in the external conditions, exerting new selection pressures. In the LTEE, for example, a fixed daily quantity of nutrients induce adaptation to this new conditions and a rise of fitness. The random noisy motion can be viewed as the result of mutations or epigenetic changes.

If the electric field is relatively small, the initial and final potential minima are relatively close to each other and the cloud described by the particle motion realizes a continuous transition between the minima (Fig. 1b).

The second situation is depicted in Fig. 1c. A double well with two distant minima is represented. The right minimum is deeper (higher fitness). This situation seems to describe cancer.

The initial (normal) state is prepared in the left well. It means that the particle starts realizing random motions from $\langle x \rangle = 0$. If the motions are of small amplitudes, the particle will remain in the left well for a long time because of the barrier preventing the transitions to the right well. Once a jump over the barrier takes place, the transition to a non zero mean value of *x* occurs. It is seen as a discontinuous transition, or a jump in the mean position of the cloud described by the random particle motions (Fig. 1d).

## Gene expression rearrangements in the LTEE

The LTEE^10^ is a formidable controlled evolution experiment with 12 *E. Coli* populations, followed for more than 60000 bacterial generations. We have studied some of the results coming from it^18,19^ with the purpose of creating a model of mutations^20^. In the present section, we use the reported GE data^21^, involving measurements in 4290 genes, in order to analyze the transition from the initial (ancestral) state to a final state at generation 20000. Data is provided for 8 harvested clones, coming from two of the twelve evolving populations in the experiment, called Ara+1 and Ara-1. 8 samples from the ancestral populations are also measured. The Ara+ and Ara- tags denote two particular mutations that were isolated from the main strain and from which all 12 populations (6 of each) were replicated, nevertheless this characteristic is not relevant for our purposes, since in effect they are simply populations that evolve independently.

The conditions stressing the bacterial populations, i.e. the scarcity of nutrients, act since the very beginning of the experiment. The transition to the new state seems to be continuous, as suggested by the observed quasi-continuous variation of fitness as a function of time^22^. We shall verify how this transition is reflected in the principal component (PC) representation and in the rearrangement of the GE distribution function.

**Figure 2:**
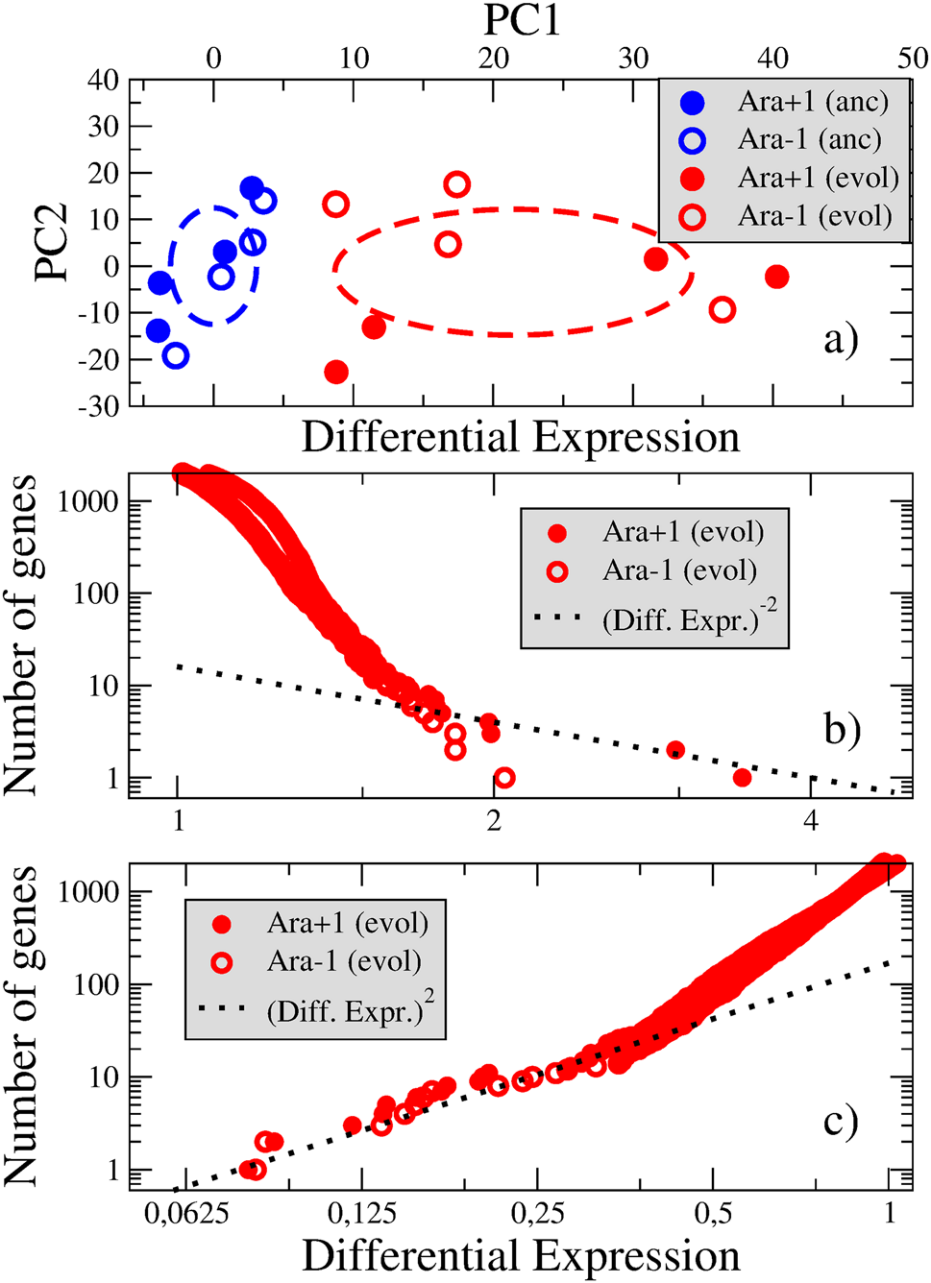
**(a)** Principal component analysis of the gene expression data in the LTEE. Samples from the ancestral (blue circles) and evolved populations (at generation 20,000, red circles) are shown. Dashed ellipses are drawn according to the standard deviations in each zone. **(b)** and **(c)** Rearrangement of the gene expression levels as a consequence of the evolution.

We show the results of the PCA in Fig. 2a. A brief description of the procedures is given in the Methods section. We define new variables, $y = \log _2{(d)}$, from which the covariance matrix is constructed. Diagonalization of the matrix leads to new coordinate axes.

The first principal component (PC1) axis, responsible for 43 % of the total data variance, seems to distinguish between the ancestral and evolved states. The coordinate $x_1$ is the projection along PC1, that is $x_1 = \mathbf {y \cdot u_1}$, where $\mathbf {u_1}$ is the normalized vector along the PC1 axis.

The mean value of the $x_1$ coordinate changes from $\langle x_1 \rangle = 0$ to $\langle x_1 \rangle = 21.44$. The mean radii of the ancestral and evolved clouds of samples, measured from the standard deviations along the PC1 axis, are 3.08 and 12.77, respectively.

Let us stress that the evolved state at generation 20000 may be seen as an intermediate stage in the transit between minima in Fig. 1. Indeed, the fitness keeps increasing at least until generation 50000^22^.

The Fig. 2b,c show the GE distribution functions. They are integrated distribution functions, that is count the number of genes with differential expression greater (lower) than a given value. Notice that the slope of the over-expression log-log curve for $1< d < 2$ (the Pareto exponent) is around -10, whereas the slope in the under-expression curve for $1> d > 1/3$ is around 4. At these points, there are changes in the exponents to values -2 and +2, respectively (the dotted lines).

There are only 4 genes in the extreme region $d > 2$ (in the Ara+1 culture), and around 20 genes in the opposite region $d < 1/3$. The total number of differentially expressed genes should be contrasted with the around 30 beneficial mutations detected at generation 20000^18,19^. Up to this point, gain of fitness is achieved mainly by turning off non active metabolic processes, i.e. by silencing the responsible genes^21^.

Summarizing the section, we may say that in the experimentally observed continuous transition in the LTEE, the initial (ancestral) and the final (evolved) states are relatively close in GE space, and the GE distribution functions of both states are also close, with only around 25 genes exhibiting significant values for the differential expression, that is a fraction of around 1/200 of the total number of genes. The latter criteria will be employed to assess the continuous character of the transition in the example studied in the next section.

## Changes in brain white matter and Alzheimer disease

The second studied example is the GE data obtained post-mortem from a cohort of patients with Alzheimer disease (AD) and nondemented controls (ND), whose ages are above 77 years. The data comes from the Aging, Dementia and TBI study by the Allen Institute^12,23^.

In the Allen study, samples are collected from four brain regions known to show neurodegeneration and be related to pathologies as a result of AD and Lewis body disease (as described in^23^): temporal and parietal neocortex (TCx and PCx), hippocampus (HIP) and white matter of the forebrain (FWM).

A general PCA picture of AD and ND samples can be found in supplementary Fig. S1. It is apparent that in the neocortex and the hippocampus, the clouds of ND and AD samples practically overlap. Samples from the white matter, on the other hand, are distributed over a wider sector in GE space, and it seems to be a clear distinction between the AD and ND zones.

Thus, below we focus on FWM. There are 47 ND and 28 AD samples, coming from different patients. The number of involved genes in the study is 50281. Notice that in the RNA-seq technology^12,13^, not only protein-coding genes are detected, but also pseudogenes, long noncoding sequences with so far unknown functions, etc. The number of genes depends on the knowledge on genes at the moment the technology is created.

**Figure 3:**
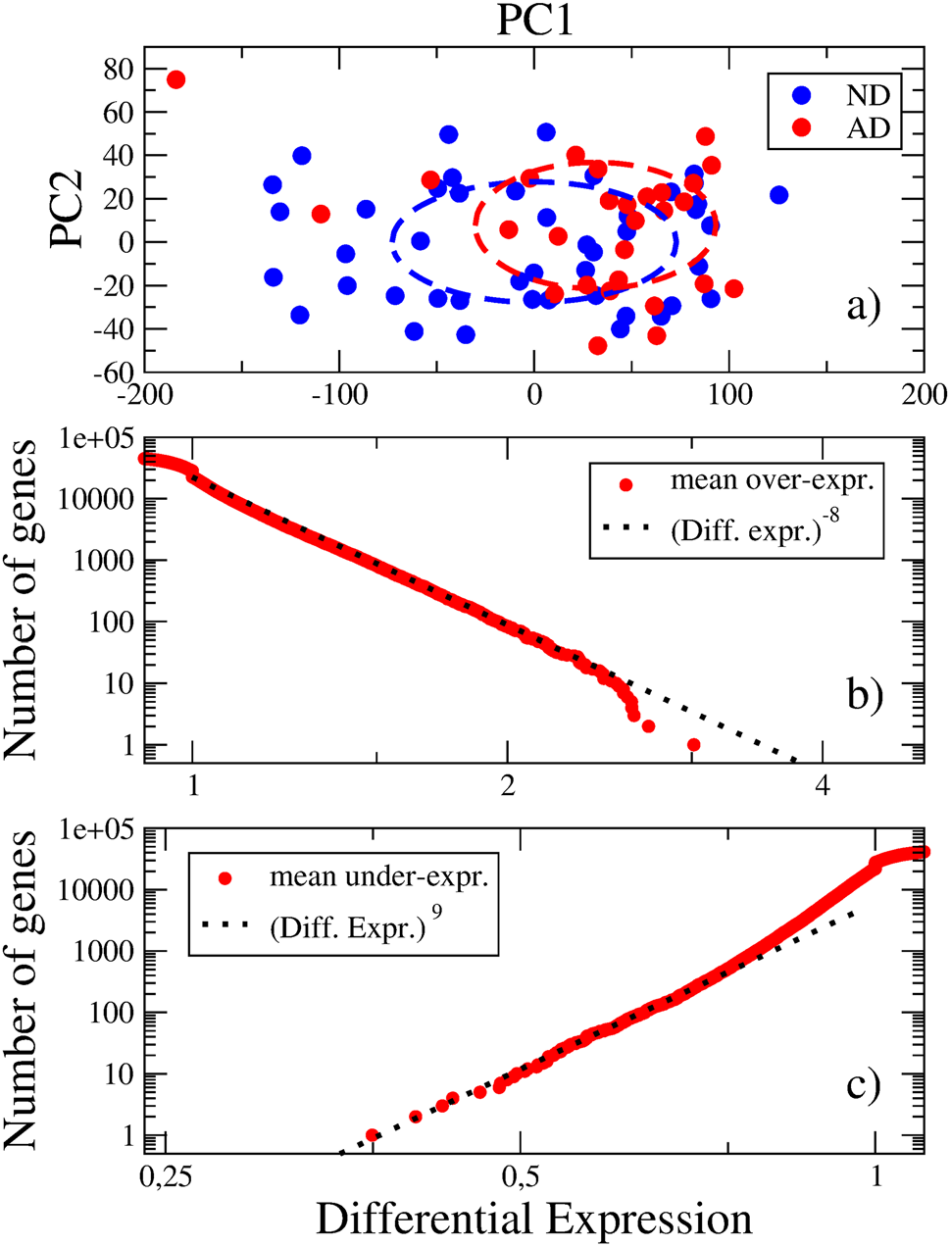
**(a)** Principal component analysis of the Allen Institute gene expression data in FWM. ND and AD samples are shown. Dashed ellipses are drawn according to the standard deviations in each zone. **(b)** and **(c)** Differential gene expression distribution functions in the AD state. Only a few dozens of genes reach significant differential expression values.

Figure 3a shows the results of the PCA of the FWM data. The PC1 axis, which accounts for 24.7 % of the total data variance, discriminates between the ND and AD states. The transition between both states is accompanied by a change from $\langle x_1 \rangle = 0$ to $\langle x_1 \rangle = 40.97$. However, the radii of the ND and AD clouds of samples are larger than the intercenter distance, that is 80.69 and 72.64, respectively. These results suggest a continuous transition in a very broad well.

**Figure 4:**
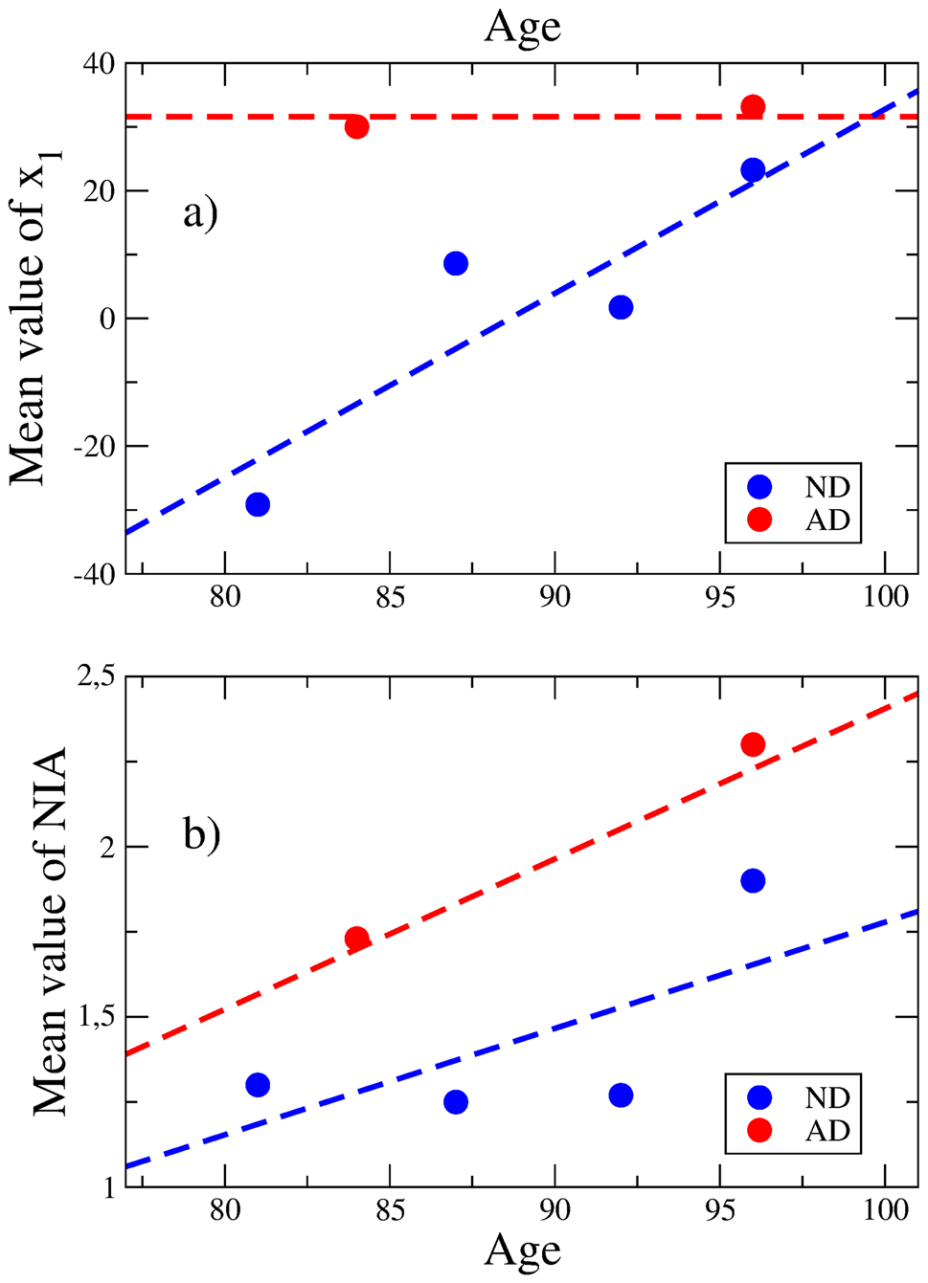
**(a)** Mean sample position along the PC1 axis as a function of age. As age increases, the ND samples experience a drift towards the AD region, which center is roughly age independent. **(b)** Age dependence of the NIA Reagan index in AD and ND samples. The BRAAK stage and CERAD score exhibit similar dependencies on age.

It is well known the role of age in AD, specially in the elderly^24^. Then, we may use age as a time variable to follow the transition. In spite of the relatively small number of samples, a linear regression analysis of the mean position $\langle x_1 \rangle$ as a function of age in ND samples, Fig. 4a, shows that $\langle x_1 \rangle \approx -287.12 + 3.24 \, age$, *P*-value $= 0.07$. In the AD samples, however, no correlation between $\langle x_1 \rangle$ and age is observed. Thus, the position of the AD zone is roughly fixed, and the cloud of ND samples shows a drift towards the AD minimum as age increases.

A better illustration of this fact comes from supplementary Fig. S2, where the probability density of ND and AD samples along the PC1 axis is compared. Four age intervals, containing roughly the same number of ND samples are defined: [77,84], [84,90], [90,95] and [95,100+] years. The total AD probability is shown in the four panels. A drift towards the AD zone as a result of aging is apparent.

Figure 4b shows the increase with age for both ND and AD samples of the NIA Reagan index for the neuropathological diagnosis of AD^25^. This may be simply interpreted as an increase of the fraction of brain microstates trapped in the AD zone.

Let us recall the physical analogy, mentioned above. The random motion of samples in GE space can not be ascribed to mutations because it is well known that the replacement rate of neurons is very low^26^. These random displacements or variations in GE space are instead related to accumulation of damage in the DNA of brain cells^27^ or to accumulation of methylation events^28,29^. Both processes are related to aging and in general lead to a decrease of tissue fitness. The roughly independence of age position of the AD zone means that this is a definite region in GE space with higher fitness, a local maximum, which holds the disease state.

The following picture of late AD progression emerges. As age increases, the fitness of brain microregions decrease and a zone of GE space representing a local maximum (the AD zone) becomes reachable. The neocortex and other brain regions are attracted earlier to this zone. The white matter, responsible for the connections and probably defining the global AD brain state, shows higher resilience. Below, we shall come back to this picture.

Figure 3b,c illustrate the rearrangement in the expression levels. The distribution function exhibits a fast decay when the differential expression departs from 1. The exponents of the Pareto laws are -8 and 9, respectively. There are around 100 genes with $d > 2$, and only around 10 genes with $d < 1/2$. The fraction of differentially expressed genes is $\sim 1/500$. The relatively small number of genes exhibiting high values of the differential expressions was stressed in the Allen Institute report^23^. We interpret it as a continuous transition between two close states: the normal aged state and the AD state. We notice that this “closeness” is only at the molecular level (not at the functional one), and that the main distinction occurs precisely in white matter, in charge of communication between brain sections.

Summarizing the section, we may say that the data on GE in the white matter of aged brains seems to support a picture of a continuous transition from the ND to the AD state motivated by a modification of the potential (the fitness distribution) at ages below 77.

## The transition from a normal tissue to a tumor

In this section, we consider a set of human tissues. In a lifetime span, the stem cells of some of them realize around 10,000 divisions^30,31^. If the tissue is in a tumor phase, an increase of the division rate is expected^32^. Thus, with respect to the number of cell divisions (generations), the data for tumor cells are comparable to that of the LTEE with bacteria.

**Table 1:** The studied cancer localizations and the main results of the section. $\langle x_1 \rangle$ is the position along PC1 of the center of the cloud of tumor samples. $R_n$ and $R_t$ are the radii, measured from the standard deviations, of the normal and tumor clouds of samples, respectively. $d_{min}$ and $d_{max}$ are the minimal and maximal values of the differential expressions, and $\upsilon _{under}$ and $\upsilon _{over}$ the Pareto exponents in the under- and over-expression regions. TCGA abbreviations for the tumors are used here, while full names are provided in the Supplementary Table 1.

Tissue	$\langle x_1 \rangle$	$R_n$	$R_t$	$d_{min}$	$\upsilon _{under}$	$d_{max}$	$\upsilon _{over}$
BLCA	140.61	57.53	34.68	0.0061	1.8	18.28	-3.5
BRCA	137.37	20.97	31.66	0.0132	2.0	70.15	-1.6
COAD	155.89	11.71	28.53	0.0032	1.6	60.41	-2.0
ESCA	138.70	64.28	35.79	0.0010	0.8	29.78	-3.0
HNSC	123.50	27.74	23.54	0.0087	1.5	45.15	-2.5
KIRC	171.80	28.70	36.00	0.0002	0.7	299.60	-1.4
KIRP	163.42	19.90	27.78	0.0001	0.8	43.16	-2.5
LIHC	134.67	20.48	45.23	0.0113	1.8	40.34	-3.0
LUAD	145.33	13.51	32.06	0.0034	1.8	41.71	-2.8
LUSC	194.49	11.62	36.65	0.0009	1.7	364.50	-1.5
PRAD	91.33	31.31	32.17	0.0439	3.2	21.81	-3.2
READ	168.05	22.90	28.81	0.0021	1.5	171.80	-1.5
STAD	136.97	27.14	43.24	0.0138	1.8	71.84	-3.5
THCA	112.54	20.02	39.85	0.0154	2.0	62.52	-2.0
UCEC	171.38	38.24	22.14	0.0054	1.7	80.45	-2.0

We analyze GE data from the TCGA^13^ for the 15 tumor localizations described in Table 1. Expression levels for 60483 genes are measured. Recall the comment above on the number of genes in the RNA-seq technology. Normal and tumor samples from different patients are recorded. Thus, we should make use of the ergodic hypothesis for the analysis of the data. We stress that a set of results coming from the PCA of this data is presented in Ref.^33^. Below, we focus on the rearrangements of GE levels.

**Figure 5:**
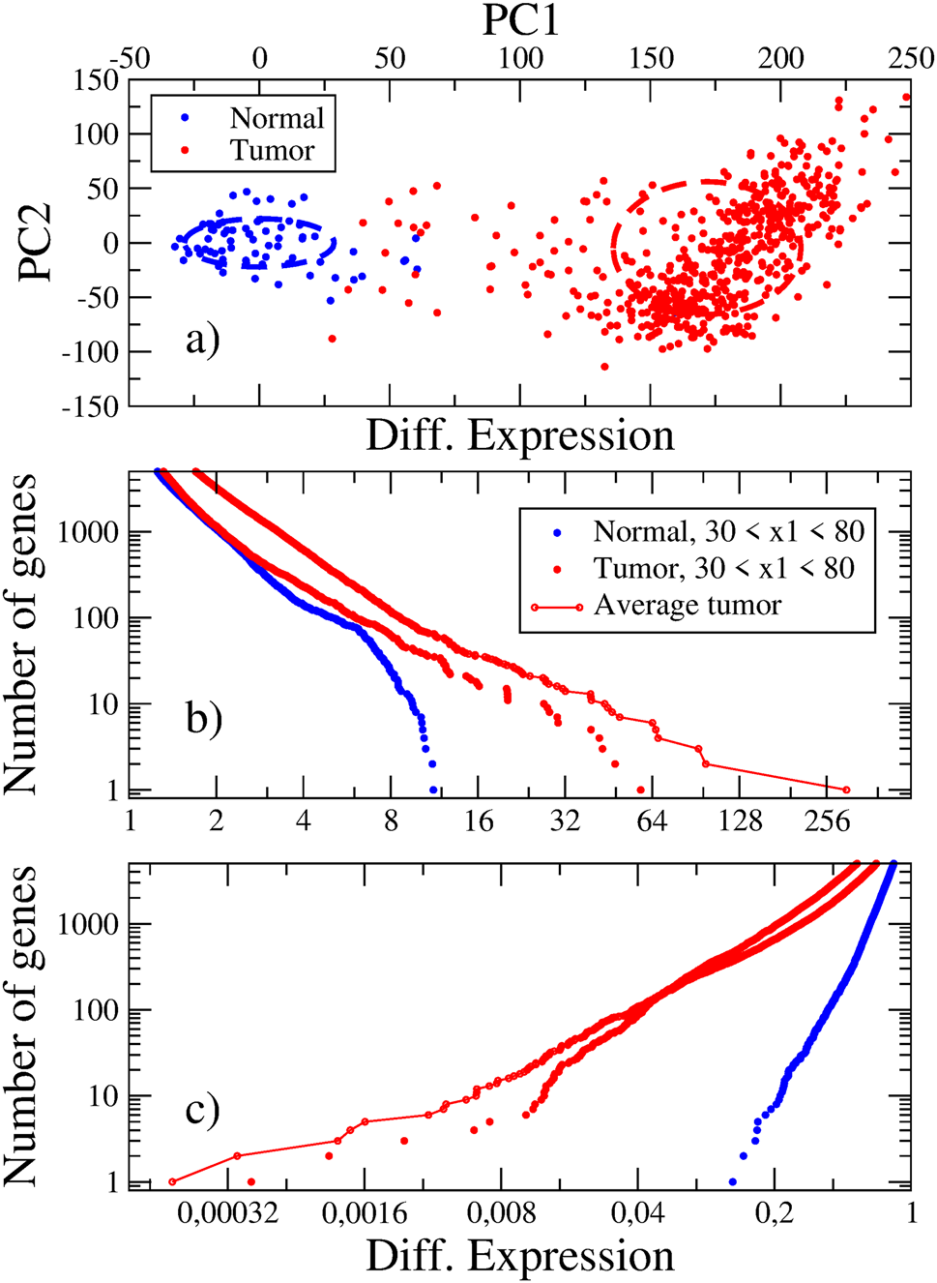
**(a)** Principal component analysis of the gene expression data in Clear Cell Kidney Cancer (KIRC). PC1 is the axis describing the progression from the normal to the tumor state. Dashed ellipses are drawn according to the standard deviations in each zone. **(b)** and **(c)** Rearrangement of the gene expression distribution function in the progression from normal tissue to tumor.

Let us consider the Kidney Clear Cell Carcinoma (KIRC) in more details. The PCA is presented in Fig. 5a. The PC1 axis, responsible for 60 % of the data variance, discriminates between normal and tumor samples. The mean value of the $x_1$ coordinate varies from $\langle x_1 \rangle =0$ to $\langle x_1 \rangle = 171.80$ in the transition from the normal to the tumor state. The radii of these regions are 28.70 and 36.00, respectively. Thus, the data suggests that there exist two distinct minima, occupying distant regions in GE space.

Notice that the number of samples in the intermediate region is scarce. This fact could be related to the common late detection of tumors^34^. Our interpretation is different. In KIRC, there are 72 normal and 739 tumor samples, large enough numbers. According to the ergodic hypothesis, the higher density of observed samples correspond to the potential wells (higher fitness regions). The deepest well seems to be the tumor state. The intermediate region $30< x_1 < 130$, supports a low-fitness barrier which prevents the transition from the normal to the tumor state. In particular, $30< x_1 < 80$ defines a coexistence region, where both normal and tumor samples are observed. Both the scarcity of samples in the intermediate region and the late detection of tumors are a consequence of the fitness landscape.

In our previous paper^35^, we have quantitatively estimated the number of available microstates in each region for a set of tumors by means of an entropy-like magnitude. This number is much greater for tumors than for the normal state. Thus, the barrier in the intermediate region is needed. Otherwise, the normal microstates could be continuously driven to the tumor region.

The progression of a normal sample to a tumor state could proceed as follows. The sample starts at a point near $x_1 = 0$ and realizes random motions due to somatic mutations, epigenetic changes or external carcinogenic factors. However, the barrier prevent the sample from leaving the normal region. Only when a jump over the barrier occurs the sample starts moving towards the tumor region.

The idea that the $x_1$ coordinate indicates progression towards the tumor region is supported by a set of facts. In paper^33^, we show in KIRC that the intermediate region is populated mainly by stage *I* tumors. In Ref.^36^ we show in PRAD (prostate cancer) that $x_1$ shows strong correlation to clinical indicators of progression, in particular tumor cellularity, that is the fraction of cancer cells in the sample.

In Fig. 5b,c, we show the distribution function for the differential expressions in the over- and under-expression regions. The average tumor curves exhibit exponents near − 1.4 and 0.7, respectively, and there are thousands of differentially expressed genes. These results favor the picture of a discontinuous normal to tumor tissue transition.

Two additional curves were added to these figures. They reflect the average distributions of normal and tumor samples in the intermediate coexistence region, and show how the rearrangement of expression levels occurs in the progression to tumors. The greatest differences between normal and tumor distributions become apparent in the under-expression region. Roughly speaking, these are genes related to homeostasis, which are silenced in the tumor state. This fact was already noticed in paper^33^. Genes may be ranked according to their contribution to the unitary vector along PC1, the axis labeling progression to cancer. In lungs, for example, the most relevant silenced gene is Surfactant Protein C, in kidney it is Uromodulin, etc. All these genes play an important role in their respective tissue homeostasis.

The results for the other tumor localizations, studied in the present paper, are summarized in Table 1. The mean value of the $x_1$ coordinate in the tumor state (for the normal state we set $\langle x_1 \rangle = 0$), the radii of the normal and tumor zones, the Pareto exponents, and the maximal and minimal reached differential expression values are given for each tissue.

We have grouped in a final supplementary Fig. S3 the distribution functions for all of the studied tumor localizations, which shows a kind of universal behavior in cancer.

Summarizing the section, we may say that the transition from a normal tissue to a tumor seems to be a discontinuous one. The differential distribution functions show very heavy tails with thousands of differentially expressed genes, around 1/10 of the total number of genes.

## Concluding remarks

We use an analogy with the motion of a particle realizing random displacements in an external potential in order to analyze the GE rearrangements in a biological system, which experiences a transition from an initial to a final state. The random motion of the particle is associated to variations in the expressions of a group of genes as a result of mutations and epigenetic events, or even damages in the DNA. The external potential is the fitness landscape.

In the LTEE, the experiment conditions induce displacement towards a new minimum, away from the initial one corresponding to the wild or ancestral genotype.

In the study concerning late onset of AD, we observe an AD zone with a definite position in GE space, and a drift of the ND clouds of samples towards the AD zone as age increases.

Both are examples of continuous transitions, motivated by a modification of the fitness landscape. This modification is well understood in the LTEE. In the AD study, on the other hand, we think that the accumulation of damages and methylation events as a result of aging is not only the reason for the random motion in GE space, but leads also to a significant reduction of fitness in the microstates. Recalling the fitness landscape in the next example, tumors, we may say that aging makes the brain microstates to move away from the normal, homeostatic zone to the low-fitness region. It seems that the AD zone is located somewhere in this region and is a kind of local maximum for the fitness, to which the ND samples are attracted.

The idea of aging as a cause for reaching the low-fitness barrier is also consistent with the increase of cancer risk with age.

The conceptualized abrupt character of the transition in cancer shows similarities with the two-stages theory (initialization-progression)^37,38^. The initialization phase is identified with the initial jump moving the microstate out of the homeostatic region. Further elaborations of this theory, i.e. Vogelstein progression in colon cancer and beyond^39,40^, indicate that there could be a sequence of steps. This is not surprising because there is a long way from the normal to the tumor regions, as shown in our calculations of distances.

We make notice that in paper^41^ we demonstrate for 8 tissues and no free parameters that the observed risks of cancer are consistent with a model of large jumps in GE space.

Continuous and discontinuous transitions are reflected in different ways in the GE distribution functions. The former corresponds to slight, whereas the latter corresponds to radical rearrangements.

We quantitatively describe the geometry of minima in GE space, and the tails of the GE distribution functions.

## Methods

The GE data corresponding to the studied examples is analyzed by means of the PCA technique. The details of the PC analysis may be found in paper^33^. We briefly sketch them in the present section.

The dimension of matrices in the Principal Component Analysis is equal to the number of genes in the data. The geometric mean is used in order to compute the average expression of the genes, where the data is slightly distorted to avoid zeroes. To this end, we added a constant to the expression (0.0001 in the LTEE data, 0.1 in the other two examples). By applying this procedure the differential expression of not statistically significant genes is regularized to one.

We define the reference expression for each gene, $e_{ref}$, by taking the mean geometric average over normal or initial state samples. Then the normalized or differential expression is defined as: $d = e / e_{ref}$. The fold variation is defined in terms of the logarithm $y = \log _2{(d)}$. Besides reducing the variance, the logarithm allows treating over- and sub-expression in a symmetrical way^33^.

Deviations and variances are measured with respect to the average over normal samples: $y = 0$. Then, the covariance matrix is written:

1 $$\begin{aligned} \mathbf {\sigma _{ij}} = \sum {y_i(s)y_j(s)}/(N_{samples} -1), \end{aligned}$$

where the sum runs over the samples, *s*, and $N_{samples}$ is the total number of samples (initial or normal plus final or disease). $y_i(s)$ is the fold variation of gene *i* in sample *s*.

By diagonalizing $\mathbf {\sigma _{ij}}$ we get the axes of maximal variance: the Principal Components (PCs). They are sorted in descending order of their contribution to the variance. PC1 accounts for a high percent of the variance, as notice in Ref.^33^ for the case of cancer. Therefore, we restrict our analysis for all cases to PC2 vs. PC1. maps.

To process the data and perform the diagonalization of $\mathbf {\sigma }$ we employ a Python routine that was ran in a node of a local cluster with 2 processors, 12 cores and 64 GB of RAM memory. More details can be found is section “Availability of data and materials”.

## Supplementary information

Supplementary Information 1.

## Data Availability

The information about the data we used, the procedures and results are integrated in a public repository that is part of the project “Processing and Analyzing Mutations and Gene Expression Data in Different Systems”: https://github.com/DarioALeonValido/evolp. The data we use for bacteria^10^ and Alzheimer^12^ are replicated in paths ../evolp/bases_external/LTEE/Gene_Expression/ and ../evolp/bases_external/Aging_Brain/ respectively. While in the case of cancer, in the path ../evolp/bases_external/TCGA/ we include the data for KIRC and provide instructions for downloading the data corresponding to any of the others cases from The Cancer Genome Atlas website^13^. To process each data set we include specific scripts for bacteria, Alzheimer and cancer in ../evolp/PCA_ecoli/, ../evolp/PCA_Alzheimer and ../evolp/PCA_cancer/ respectively. There is also an additional script located in the last of the previous directories where we collect the routines we implemented for the Principal Component Analysis method.
