# Supplementary material for: Gene expression rearrangements denoting changes in the biological state
Source: Sci Rep. 2021 Apr 19;11:8470. doi: 10.1038/s41598-021-87764-0 (PMC8055689; doi:10.1038/s41598-021-87764-0)
Supplement: Supplementary file 1 — Supplementary Information 1. [file 41598_2021_87764_MOESM1_ESM.pdf]

Supplementary Information for: "Gene expression rearrangements denoting changes in the biological state"

Augusto Gonzalez[1,2], Joan Nieves[3], Dario A. Leon[4,2], Maria Luisa Bringas Vega[1,5], and Pedro Valdes Sosa[1,5,*]

[1]University of Electronic Science and Technology, 610051, Chengdu, People Republic of China
[2]Institute of Cybernetics, Mathematics and Physics, 10400, Havana, Cuba
[3]Faculty of Physics, University of Havana, 10400, Havana, Cuba
[4]University of Modena & Reggio Emilia, 41125, Modena, Italy
[5]Cuban Neurosciences Center, 11600, Havana, Cuba
[*]pedro.valdes@neuroinformatics-collaboratory.org

ABSTRACT

We provide supplementary tables and figures in support of the main article.

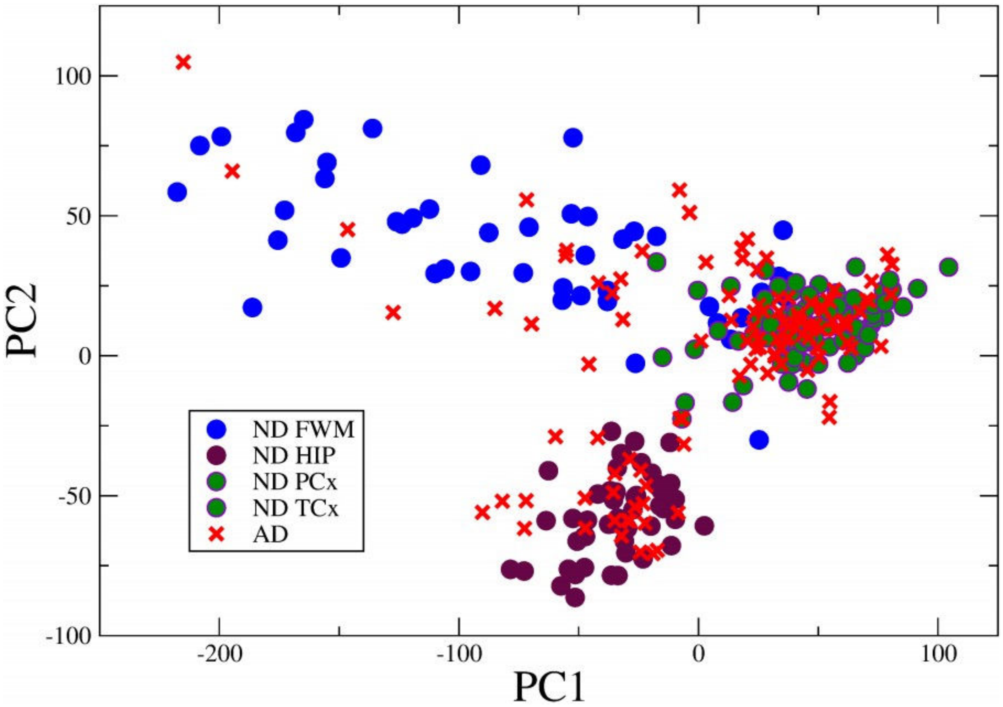

Figure S1. Principal component analysis of the Allen Institute GE data on AD. ND and AD samples are shown in each of the four studied brain regions: FWM, HIP, PCx and TCx. The best separation between ND and AD states occurs in FWM, in the other three regions the clouds of ND and AD samples practically overlap.

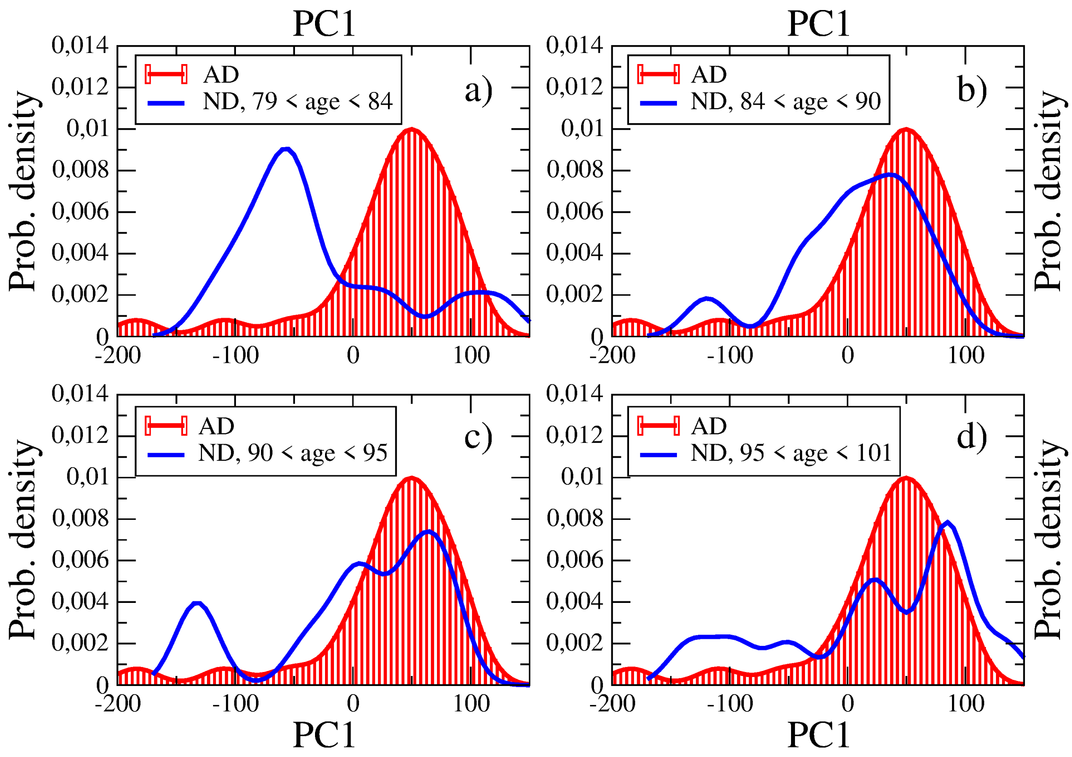

Figure S2. Probability density of ND and AD samples along the PC1 axis. Each panel refers to an age interval for ND samples. The AD probability, which is roughly age independent, is shown in the four panels.

Abbreviation	Cancer type
BLCA	Bladder Urothelial Carcinoma
BRCA	Breast invasive carcinoma
COAD	Colon adenocarcinoma
ESCA	Esophageal carcinoma
HNSC	Head and and neck squamous cell carcinoma
KIRC	Kidney clear cell carcinoma
KIRP	Kidney papillary cell carcinoma
LIHC	Liver hepatocellular carcinoma
LUAD	Lung adenocarcinoma
LUSC	Lung squamous cell carcinoma
PRAD	Prostate adenocarcinoma
READ	Rectum adenocarcinoma
STAD	Stomach adenocarcinoma
THCA	Thyroid carcinoma
UCEC	Uterine corpus endometrial carcinoma

Table S1. TCGA abbreviations for the set of studied cancer types.

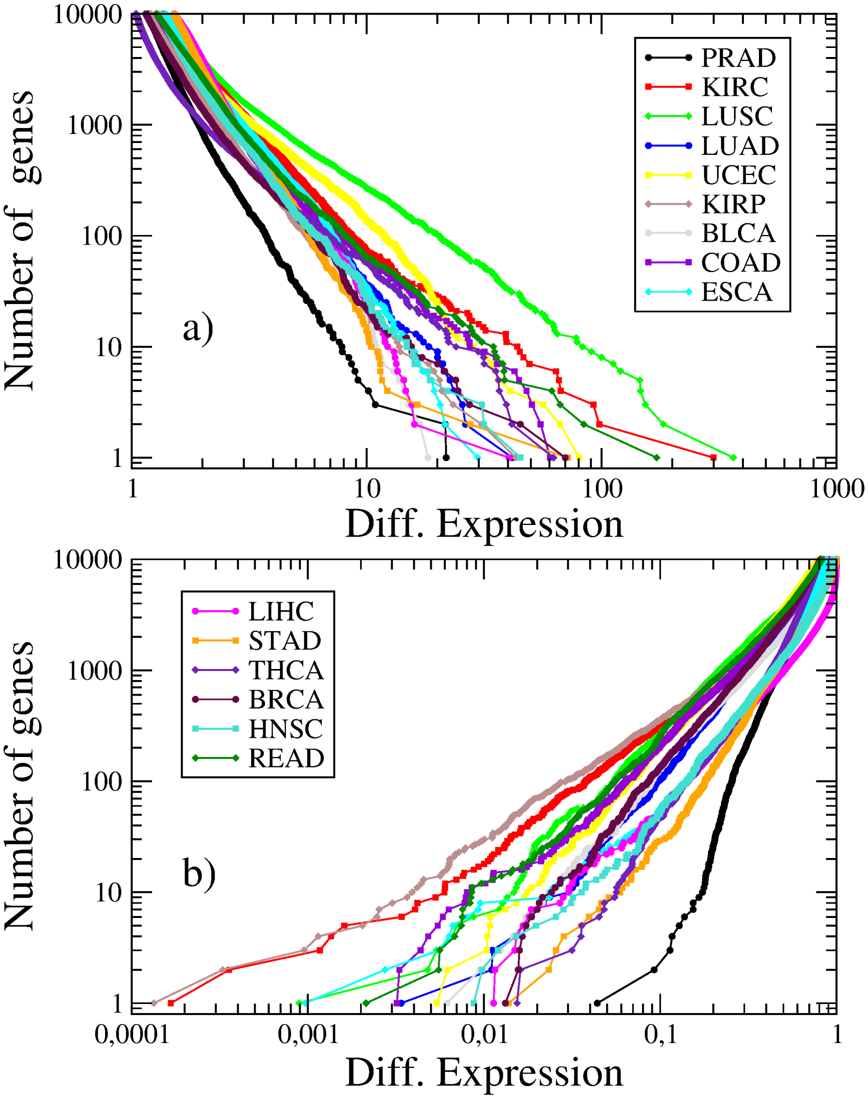

Figure S3. The differential gene expression integrated distribution functions for 15 tumor localizations in the TCGA. Pareto exponents between -1.4 and -3.5 in the over-expression tails, and between 0.7 and 2.0 in the under-expression tails are observed.
